# Does a child's mid‐upper arm circumference‐for‐age *z*‐score represent another nutritional indicator of childhood malnutrition status?

**DOI:** 10.1111/mcn.13404

**Published:** 2022-07-12

**Authors:** Md Ahshanul Haque, Nuzhat Choudhury, S. M. Tanvir Ahmed, Fahmida Dil Farzana, Mohammad Ali, Farina Naz, Mohammad Jyoti Raihan, Sheikh Shahed Rahman, Towfida Jahan Siddiqua, Abu Syed Golam Faruque, Tahmeed Ahmed

**Affiliations:** ^1^ Nutrition and Clinical Services Division, icddr,b Dhaka Bangladesh; ^2^ Save the Children Bangladesh Dhaka Bangladesh

**Keywords:** child nutrition, epidemiology, food security, health policy, maternal nutrition, wasting

## Abstract

Child wasting is defined as a weight‐for‐height/length *z*‐score (WLZ/WHZ) < −2, and this indicator of nutritional status is used worldwide. However, a precise measurement is required for the assessment of a child's nutritional status, which may not always be possible due to expensive instruments, especially in poor resource settings. In some instances, mid‐upper arm circumference‐for‐age *z*‐score (MUACZ) is also being used for screening purposes, which is a simple and useful nutritional indicator. The objective of this paper is to identify the optimal cut‐off point for the MUACZ to identify wasted children, and also to determine if the same factors are associated with MUACZ and wasting. Data were derived from the *Suchana* evaluation data. The optimal cut‐off value was estimated via receiver operating characteristic (ROC) curve analysis using acute malnutrition as a gold standard with maximum sensitivity and specificity. Multiple logistic regression was used to assess the associated factors with the MUACZ. Using the gold standard indicator of nonwasting (WLZ ≥ −2), a positive outcome, the optimal cut‐off point for the MUACZ was −1.27. The area under the ROC curve was 0.88, indicating that the model had a power of 88% to differentiate between the positive and negative classes. It implies that a child's MUACZ was correlated with WLZ, and a MUACZ below −1.27 appeared to accurately identify wasting among children aged 3–23 months. MUACZ < −1.27 might be another useful indicator of childhood wasting than a WLZ < −2.

## Key messages

- We calculated the cut‐off point for a child's mid‐upper arm circumference‐for‐age *z*‐score (MUACZ) that MUACZ below −1.27 appears to accurately identify acute malnutrition among children aged 3–23 months with the highest sensitivity and specificity.
- Determinants of a child's MUACZ < −1.27 were estimated.
- Measurement of MUAC would be especially helpful when large numbers of children need to be screened quickly, particularly in humanitarian aid settings.
- MUACZ < −1.27 may represent a more useful indicator of childhood malnutrition than a weight‐for‐length *z*‐score < −2.

## 1 INTRODUCTION

Childhood wasting, a major public health concern, is defined as a weight‐for‐height/length *z*‐score (WLZ/WHZ) < −2. The WLZ/WHZ is widely used globally as an indicator of nutritional status in children (Bari et al., 2019). If not identified in a timely manner, childhood acute malnutrition can be further deteriorated in severity. Growth monitoring and promotion programmes are widely conducted in low‐ and middle‐income countries (LMICs) to assess nutritional status by measuring children's weight and height at health care centres (Liu et al., 2017). However, these measurement tools have several drawbacks and practical limitations for rapid assessment, especially for debilitated, acutely ill or disabled children (Haque et al., 2021). Furthermore, anthropometric techniques are prone to inaccuracies, which could occur from inadequate personnel training, for instance. In a previous cohort study, an anthropometric measurement requirement, such as observer training and data collection supervision, was shown to be more difficult to implement than first anticipated. In a situation where qualified supervisors and constant training are not available, the greater necessity for sustained training would jeopardize its effectiveness (Sicotte et al., 2010). Moreover, it can be difficult to accurately measure the length and weight of children under 24 months old. Another limitation is that the instruments used to measure length and weight might not always be precise. Moreover, high‐precision instruments are very expensive in resource‐constrained settings or population‐based surveys (Haque et al., 2021). Mid‐upper arm circumference (MUAC), a common anthropometric measurement, is being used especially in emergency and crisis settings to assess the nutritional status of children, which might be an alternative option to measure WLZ/WHZ (Briend et al., 1987; Sultana et al., 2015; World Health Organization (WHO) Working Group, 1986). Children with a MUAC of less than 11 cm are categorized as malnourished (Bari et al., 2019; Briend et al., 1987; Fernández et al., 2010; Hossain et al., 2017; Takyi et al., 2020). Literature suggested several cut‐off values for MUAC, which range from <11 to 12.5 cm (WHO, 2009).

However, the MUAC is highly correlated with age and sex. Thus, an age‐ and sex‐specific indicator might be more reliable. Several kinds of literature also suggested that the MUAC‐for‐age *z*‐score (MUACZ) could be an independent indicator of child malnutrition, which is less expensive and also gives similar estimates as WLZ (Becker et al., 2014; Sadler et al., 2011; Stephens et al., 2018). Therefore, MUACZ may represent an alternative indicator for defining a child's nutritional status. MUACZ is calculated as a *z*‐score scale using the 2006 WHO Standards for Children, as (observed MUAC value − average value of the reference population)/standard deviation value of reference population (Custodio et al., 2018; Haque et al., 2019; Miller et al., 2019; Onis & Bloessner, 1997). However, as far as we are aware, no large‐scale study has been conducted to identify the optimal MUACZ cut‐off point with the highest sensitivity and specificity in the context of Bangladesh.

Furthermore, for the establishment of using MUACZ, it is also needed to compare if MUACZ‐associated factors are the same as factors that are associated with WLZ. In this regard, there are several factors, for example, sex, age, morbidity, infant and young child feeding practices, maternal age, health care practices, unimproved place of delivery, dietary diversity, household food insecurity, socioeconomic status, unimproved water and sanitation, unimproved household materials and the level of education of the household head (Choudhury et al., 2017; Khan & Kraemer, 2009; MAL‐ED Network Investigators, 2017; Platts‐Mills et al., 2017), which are associated with child nutritional status (Choudhury et al., 2017; Khan & Kraemer, 2009; MAL‐ED Network Investigators, 2017; Platts‐Mills et al., 2017). These factors were also found similar while MUACZ was used as a continuous variable. In the programmatic aspect, it is always desirable to get one cut‐off point, which might be more user‐friendly. Thus, the objective of this study is to identify the optimal cut‐off point for MUACZ to classify with wasting and to examine the associated factors if they are similar to WLZ.

## 2 METHODS

### 2.1 Study design and population

The large‐scale *Suchana* development programme was conducted among the most susceptible households in vulnerable villages in the Sylhet division, located in the northeast region of Bangladesh. Data were obtained for this paper from the *Suchana* evaluation data (Choudhury et al., 2020; Haque et al., 2020). *Suchana* evaluation was a pre–post randomized cluster design with two cross‐sectional surveys. Baseline data were collected from mother–child dyads in the year 2016, whereas the endline data were collected in the year 2019. The age of the children during enrolment was 0–23 months. For endline data collection, new mother–child dyads were recruited where the age of the children was the same as that during enrolment.

### 2.2 Sample size

The sample size was calculated for this study based on the main objective of *Suchana* evaluation. The objectives of *Suchana* programme were to reduce childhood stunting and improve exclusive breastfeeding and a minimum acceptable diet. The calculated sample size was 16,162 (baseline: 5440; endline: 10,722) (Choudhury et al., 2020). We excluded 38 cases from the database due to anthropometry measurement errors. We also excluded 1281 cases for the children aged less than 3 months, owing to MUACZ being not applicable for children aged 0–3 months. Finally, we analysed 14,843 cases for this paper.

### 2.3 Data collection

Android tablets complemented by custom‐developed Java software were employed for data collection during the surveys. The mobile‐based data collection process reduced the data entry burden, as the data were entered at the interviewer level and the records were uploaded to a server at the icddr,b using the built‐in internet connectivity of the devices. This allowed the data analysis team to review the consistency of the data every day. The Java software‐based electronic questionnaire was designed as survey forms in both Bangla and English languages, which were interchangeable at any time during the data collection process. The enumerators used the Bangla form on the PDA while interviewing the respondents and recording anthropometric measurements. A standard operating procedure was provided to all staff. Editing and updating,  range, consistency, frequency and duplication checks and cross‐tabulation of the data were regularly performed using the data entry period. Unusual observations were discussed and resolved on a daily basis. A secure web‐based data management system was used to manage the data. When data collection was complete, the data were transferred into Stata software release 14 (StataCorp) to define the variable and value labels (Haque et al., 2022).

SECA 874 weight scales with an accuracy of 1 g were used to measure maternal and child weight. The mother was asked to remove all *jewellery* and accessories and wear minimum culturally acceptable clothing before standing on the weight scale. Then, the mother was asked to hold her child and stand on the scale. Two consecutive readings were taken; if the difference between readings was more than 50 g, a third measurement was taken and then averaged. Then, the mother was requested to stand alone on the weight scale and the weighing process was repeated. The difference in weight between the mother and child and mother was calculated automatically by the survey software and logged as the weight of the child. SECA 416 infantometers with a precision of 0.1 cm were used to measure the children's length; a third measurement was taken if the first two consecutive measurements differed by more than 2 cm. MUAC is measured at the mid‐point between the tip of the shoulder and the tip of the elbow (olecranon process and acromion process). Child's age and sex‐specific anthropometry indicators, such as length‐for‐age, weight‐for‐age, weight‐for‐length and MUACZ, were calculated using the 2006 WHO Standards for Children, as (observed anthropometry value − average value of the reference population)/standard deviation value of reference population (Custodio et al., 2018; Haque et al., 2019; Miller et al., 2019; Onis & Bloessner, 1997). Child's weight in kilogram, length in centimetre, MUAC in centimetre, birth date and survey data were entered in the WHO Anthro (version 3.2.2) software to calculate the *z*‐scores. Children were defined as stunted if their length‐for‐age is less than minus two standard deviations (LAZ < −2), wasted if their weight‐for‐length is less than minus two standard deviations (WLZ < −2) and underweight if their weight‐for‐age is less than minus two standard deviations (WAZ < −2) (Haque et al., 2021).

### 2.4 Outcome variable

The primary outcome variable was children's MUACZ. The optimal cut‐off value was estimated through receiver operating characteristic (ROC) curve analysis as well as Youden's index using acute malnutrition as the gold standard with maximum sensitivity and specificity. A child was labelled as wasted if his/her MUACZ is less than the optimal cut‐off point.

### 2.5 Independent variables

**Figure 1:**
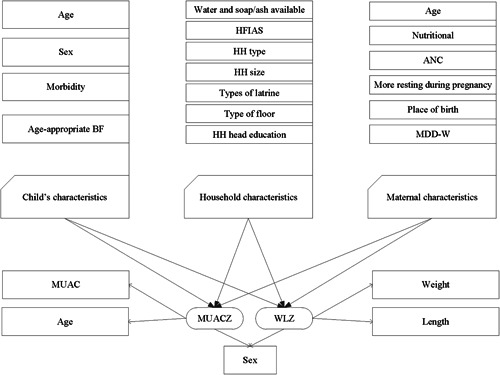
Conceptual framework of factors that affect childhood malnutrition. ANC, antenatal care; BF, breastfed; HFIAS, household food security access scale; HH, household; MDD‐W, minimum dietary diversity for women; MUACZ, mid‐upper arm circumference‐for‐age *z*‐score; WLZ, weight‐for‐length *z*‐score.

A list of independent variables was finalized through a literature review and descriptive analysis. The conceptual framework for this study is shown in Figure 1. The children's characteristics were age, sex, experience of any illness in the last 15 days and age‐appropriate breastfeeding. Age‐appropriate breastfeeding was defined as infants 0–5 months of age who received only breast milk during the previous day, and children 6–23 months of age who received breast milk, as well as solid, semisolid or soft foods, during the previous day. Maternal characteristics were age, MUAC < 24 cm as nutritional status, at least four antenatal care (ANC) visits by a skilled service provider, resting more than usual during last pregnancy (Haque et al., 2022), delivery in a health care facility, experience of domestic violence (Haque et al., 2020) and minimum dietary diversity for women (MDD‐W). In our questionnaire, we asked women whether they rested more than usual while pregnant with their most recent child. We had three options: (i) more, (ii) as before and (iii) less. ‘More’ was considered as the indicator of ‘resting more than usual during the last pregnancy’. Experience of domestic violence was defined as the husband threatening to divorce his wife or marry another woman, or if the husband or another family member had verbally or physically abused the participant at any point of time since their marriage. MDD‐W was defined as mothers consuming at least five food groups, that is, the number of unique food items consumed in the last 24h by the target mother. The 10‐food group scoring system was based on the FANTA III guideline (Daniels et al., 2009; FAO and FHI 360, 2016). The household characteristics were the water and soap/ash available within 30 feet of the toilet structure, Household Food Insecurity Access Scale (HFIAS), household type (poor/very poor), household size, type of latrine, type of floor and household head education. The HFIAS was determined using the ‘Food and Nutrition Technical Assistance's Guideline’ and categorized as food secure, mildly food insecure, moderately food insecure or severely food insecure (Coates et al., 2007). The household type (poor and very poor) was determined by a wealth ranking exercise, which was carried out to identify potential target beneficiary households (Choudhury et al., 2020).

### 2.6 Statistical analysis

Data were visualized using histograms, bar diagrams, pie charts and scatter plots, as appropriate. Descriptive statistics were used to summarize data (frequency and proportion for categorical variables, mean and standard deviation for quantitative variables). The descriptive analyses focused on the indicator MUACZ.

Pearson's correlation coefficients (*r*) were calculated to assess the linear relationships between children's WLZ and MUACZ. ROC curve analysis for MUACZ was performed based on nonwasting (WLZ ≥ −2); a healthy child was defined as a positive outcome (Sultana et al., 2015). The area under ROC curves was estimated to compare the aggregated classification performance of new indicators; the optimal MUACZ cut‐off point was identified based on maximum sensitivity and specificity. In general, an AUC of 0.5 suggests no discrimination, 0.7 –0.8 is considered acceptable, 0.8–0.9 is considered excellent and more than 0.9 is considered outstanding (Mandrekar, 2010). Our hypothesis is that the values of sensitivity and specificity would be close to 80%. We also applied Youden's index to calculate the empirical optimal cut‐off point (Sultana et al., 2015).

Finally, we explored the associations between MUACZ and other indicators associated with childhood malnutrition in previous studies. Simple logistic regression was used to assess the bivariate relationships between MUACZ as a categorical variable (MUACZ ≥ cut‐off point denotes healthy and MUACZ < cut‐off point denotes malnourished) and the other independent variables. Multiple logistic regression was employed to identify the factors independently associated with the outcome indicator after adjusting for *union* (i.e., the smallest unit of the *Suchana* study areas) as a cluster. The variables were included in multiple regression analysis by stepwise forward selection if their *p* < 0.25 (Bursac et al., 2008); some indicators, such as age, sex and other relevant variables were included regardless of their *p *value. In the final model, *p* < 0.05 was considered the significance level. We also performed the same analysis for MUAC < 12.5 cm, MUAC < −2 and WLZ < −2 to compare with a new indicator.

## 3 RESULTS

**Figure 2:**
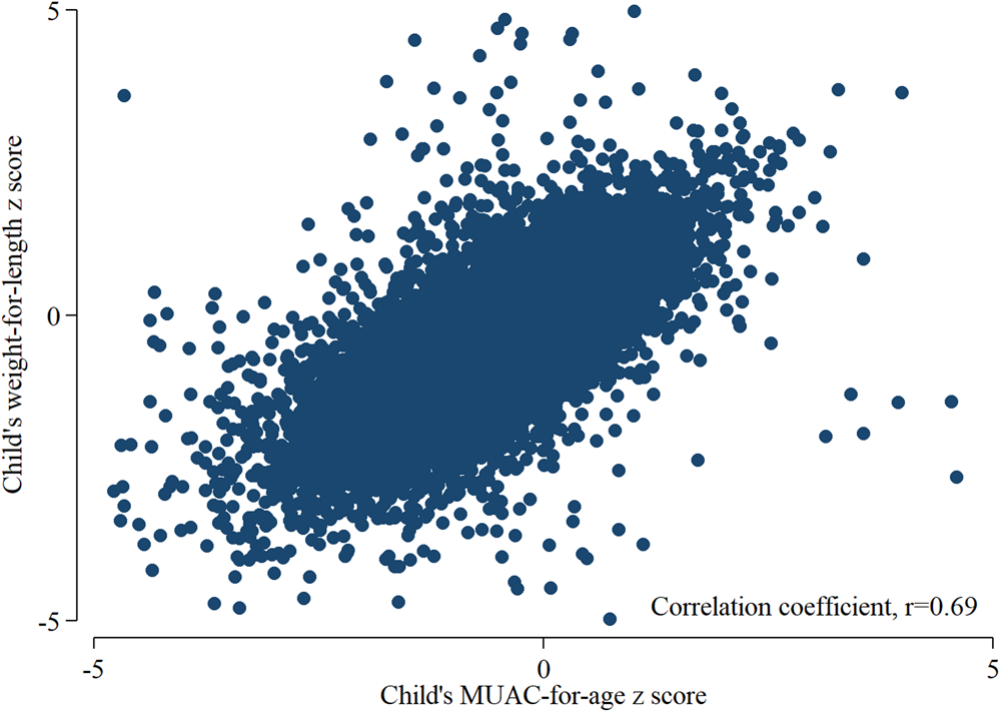
Scatterplot illustrating the relationship between children's weight‐for‐length *z*‐scores and mid‐upper arm circumference *z*‐scores.

**Figure 3:**
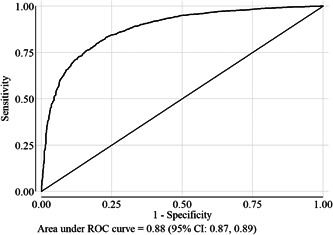
Screening test of WLZ (<−2) by MUACZ based on area under ROC curve analysis; a nonwasting child (WLZ ≥ −2.0 and MUACZ ≥ −1.27) was defined as a positive outcome in the ROC curve analyses. MUACZ, mid‐upper arm circumference‐for‐age *z*‐score; ROC, receiver operating characteristic; WLZ, weight‐for‐length *z*‐score.

A total of 14,843 children (3–23 months) data was used for this analysis. Children below 3 months were not included in this analysis because the WHO Anthro Application calculated the MUACZ for only the age group between 3 months and 5 years. The general characteristics of the children, women and households are presented in Table 1. Around 25.0% of children had a MUACZ < −1.27, whereas the prevalence of wasting was 7.5%, stunting was 44.0% and underweight was 28%. The correlation coefficient (Figure 2) between WLZ and MUACZ was very high (*r* = 0.69). In the ROC curve analyses using nonwasting (WLZ ≥ −2) as a gold standard indicator and a healthy child as a positive outcome, the optimal cut‐off point for MUACZ was −1.27. The area under the ROC curve was 0.88 (95% confidence interval [CI]: 0.87, 0.89), indicating that this cut‐off had an 88% probability of differentiating between the wasting and nonwasting outcome (Figure 3).

**Table 1:** General characteristics of the subjects

Indicators, % (*n*)	Female children	Male children	All
*Children's characteristics*			
Nutritional status			
MUAC‐for age *z*‐score < −1.27	22.81 (1667)	26.28 (1980)	24.57 (3647)
MUAC < 12.5 cm	12.83 (938)	7.27 (548)	10.01 (1486)
MUAC‐for age *z*‐score < −2	6.53 (477)	8.53 (643)	7.55 (1120)
Childhood wasting	6.33 (463)	8.64 (651)	7.51 (1114)
Childhood stunting	41.22 (3013)	46.72 (3520)	44.01 (6533)
Childhood underweight	25.69 (1878)	29.52 (2224)	27.64 (4102)
Child's length‐for‐age *z*‐score^a^	−1.78 (1.12)	−1.91 (1.22)	−1.85 (1.17)
Child's weight‐for‐age *z*‐score^a^	−1.37 (1.05)	−1.43 (1.12)	−1.4 (1.09)
Child's weight‐for‐length *z*‐score^a^	−0.55 (1.00)	−0.59 (1.09)	−0.57 (1.05)
Child's MUAC‐for‐age *z*‐score^a^	−0.63 (0.92)	−0.67 (0.99)	−0.65 (0.96)
Age (months)			
Child's age: <6 months	12.01 (878)	11.77 (887)	11.89 (1765)
Child's age: 6–11 months	24.94 (1823)	23.41 (1764)	24.17 (3587)
Child's age: 12–23 months	63.05 (4608)	64.81 (4883)	63.94 (9491)
Experienced any illness in the last 15 days	46.7 (3413)	49.04 (3695)	47.89 (7108)
Age‐appropriate BF	89.05 (6509)	90.32 (6805)	89.7 (13314)
*Maternal characteristics*			
Maternal age (years)			
Maternal age: <25 years	30.88 (2257)	31.91 (2404)	31.4 (4661)
Maternal age: 25–30 years	30.5 (2229)	30.45 (2294)	30.47 (4523)
Maternal age: ≥30 years	38.62 (2823)	37.64 (2836)	38.13 (5659)
Maternal MUAC < 24 cm	52.17 (3813)	54.33 (4093)	53.26 (7906)
At least four ANC visits	23.64 (1728)	23.36 (1760)	23.5 (3488)
More resting during pregnancy	35.64 (2605)	36.24 (2730)	35.95 (5335)
Delivery in a health care facility	26.67 (1949)	30.69 (2312)	28.71 (4261)
Water and soap/ashwere available within 30 feet of the toilet structure	42.03 (3072)	42.37 (3192)	42.2 (6264)
At least 5 out of 10 defined foods	37.98 (2776)	37.6 (2833)	37.79 (5609)
*Household characteristics*			
Household food security status			
Food secure	19.62 (1434)	20.42 (1538)	20.02 (2972)
Mildly food insecure	13.96 (1020)	13.95 (1051)	13.95 (2071)
Moderately food insecure	45.79 (3347)	45.17 (3403)	45.48 (6750)
Severely food insecure	20.63 (1508)	20.46 (1541)	20.54 (3049)
Household type			
Poor	53.71 (3926)	53.07 (3998)	53.39 (7924)
Very poor	46.29 (3383)	46.93 (3536)	46.61 (6919)
Household size ≤ 4	24.91 (1821)	24.49 (1845)	24.7 (3666)
Improved latrine	41.21 (3012)	40.54 (3054)	40.87 (6066)
Improved floor	14.19 (1037)	14.69 (1107)	14.44 (2144)
Head of the household with no schooling	45.15 (3297)	43.97 (3310)	44.55 (6607)

^a^ Mean (SD).

**Figure 4:**
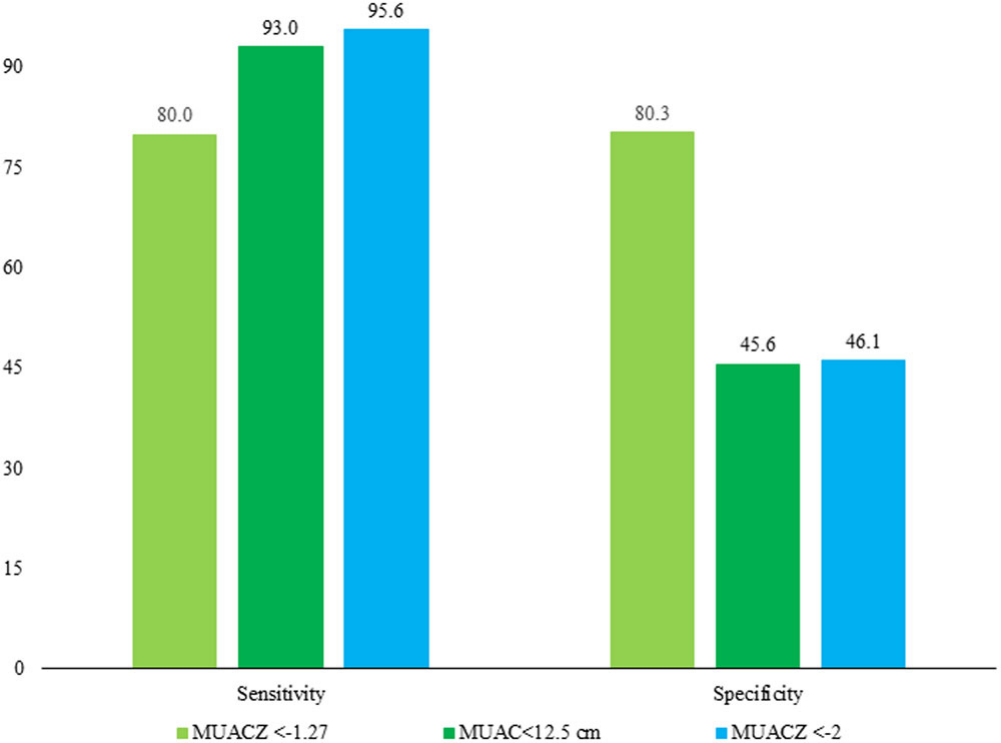
Sensitivity and specificity of a child's MUACZ < −1.27, MUAC < 12.5 cm and MUACZ < −2 as new indicators of malnutrition versus wasting; nonwasting was defined as a positive outcome in the sensitivity and specificity analyses. MUACZ, MUAC‐for‐age *z*‐score.

The sensitivity for this cut‐off point was 80.0%, indicating that 80.0% of children classified as healthy based on a MUACZ ≥ −1.27 would be defined as nonwasting based on a WLZ ≥ −2. The empirical optimal cut‐off point using Youden's index has given the same value. Moreover, the specificity for this cut‐off was 88.3%, indicating that 88.3% of children were classified as unhealthy based on a MUACZ < −1.27 would be defined as wasting based on a WLZ < −2. The sensitivity and specificity values for the optimal MUACZ cut‐off for wasting based on WLZ are presented in Figure 4. We also calculated the sensitivity and specificity for the cut‐off point for MUAC values < 12.5 cm and MUACZ values < −2, which are given in Figure 4.

We also determined the factors associated (Table 2) with a MUACZ < −1.27 as an indicator of malnourished children using multiple logistic regression; adjusted odds ratios (aORs) were estimated to examine the strength of the associations. In terms of the children's characteristics, ages of 6‒11 months [aOR: 1.22 (95% CI: 1.08, 1.37); *p* < 0.001] and 12‒23 months [aOR: 1.58 (95% CI: 1.41, 1.77); *p* < 0.001] were associated with a MUACZ < −1.27 as an indicator of malnourished children compared with children aged <6 months. Moreover, male sex [aOR: 1.21 (95% CI: 1.12, 1.30); *p* < 0.001], experience of any illness in the last 15 days [aOR: 1.19 (95% CI: 1.11, 1.28); *p* < 0.001] and children not practicing age‐appropriate breastfeeding [aOR: 1.20 (95% CI: 1.05, 1.38); *p* = 0.009] were also associated with the outcome of a MUACZ < −1.27 as an indicator of malnourished children.

**Table 2:** Factors associated with a child's MUACZ < −1.27 as new indicators of childhood malnutrition, mid‐upper arm circumference < 12.5 cm, MUACZ < −2 and weight‐for‐length *z*‐score < −2 as an indicator of childhood acute malnutrition

	WLZ<−2		MUACZ < −1.27		MUAC < 12.5 cm		MUACZ < −2	
Indicators	Adjusted OR	*p *value	Adjusted OR(95% CI) (95% CI)	*p *value	Adjusted OR (95% CI)	*p *value	Adjusted OR (95% CI)	*p *value
Age (months)								
Child's age: <6 months	Reference		Reference		Reference		Reference	
Child's age: 6–11 months	1.33 (1.05, 1.69)	0.020	1.22 (1.08, 1.37)	0.001	0.41 (0.35, 0.49)	<0.001	0.99 (0.79, 1.24)	0.939
Child's age: 12–23 months	1.78 (1.40, 2.27)	<0.001	1.58 (1.41, 1.77)	<0.001	0.24 (0.21, 0.28)	<0.001	1.24 (1.03, 1.50)	0.025
Child's sex								
Female	Reference		Reference		Reference		Reference	
Male	1.39 (1.24, 1.56)	<0.001	1.21 (1.12, 1.30)	<0.001	0.51 (0.45, 0.58)	<0.001	1.33 (1.17, 1.51)	<0.001
Experienced any illness in the last 15 days								
No	Reference		Reference		Reference		Reference	
Yes	1.23 (1.08, 1.40)	0.001	1.19 (1.11, 1.28)	<0.001	1.29 (1.14, 1.46)	<0.001	1.27 (1.12, 1.44)	<0.001
Age‐appropriate breastfeeding								
Yes	Reference		Reference		Reference		Reference	
No	1.23 (1.00, 1.52)	0.049	1.20 (1.05, 1.38)	0.009	1.17 (0.99, 1.39)	0.068	1.28 (1.05, 1.57)	0.016
Maternal age (years)								
Age < 25	Reference		Reference		Reference		Reference	
Age ≥ 25	1.17 (1.01, 1.36)	0.040	1.12 (1.02, 1.23)	0.016	1.06 (0.93, 1.21)	0.400	1.23 (1.04, 1.46)	0.014
Maternal MUAC (cm)								
MUAC ≥ 24	Reference		Reference		Reference		Reference	
MUAC < 24	1.64 (1.41, 1.92)	<0.001	1.73 (1.59, 1.88)	<0.001	1.96 (1.76, 2.18)	<0.001	1.81 (1.60, 2.05)	<0.001
At least four ANC visits by a skilled service provider								
Yes	Reference		Reference		Reference		Reference	
No	1.07 (0.89, 1.28)	0.479	1.15 (1.03, 1.28)	0.013	1.32 (1.13, 1.54)	<0.001	1.39 (1.15, 1.68)	0.001
More resting during pregnancy								
Yes	Reference		Reference		Reference		Reference	
No	1.19 (1.02, 1.38)	0.024	1.12 (1.02, 1.23)	0.021	1.23 (1.09, 1.38)	0.001	1.22 (1.05, 1.41)	0.009
Delivery in a health care facility								
Yes	Reference		Reference		Reference		Reference	
No	1.14 (0.97, 1.33)	0.116	1.23 (1.12, 1.35)	<0.001	1.17 (1.01, 1.37)	0.042	1.13 (0.96, 1.34)	0.134
MDD‐W								
At least five groups	Reference		Reference		Reference		Reference	
Less than five groups	1.13 (0.99, 1.28)	0.060	1.11 (1.02, 1.22)	0.020	1.11 (0.98, 1.27)	0.095	1.21 (1.04, 1.41)	0.015
Water and soap/ash were available within 30 feet of the toilet structure								
Yes	Reference		Reference		Reference		Reference	
No	1.18 (1.02, 1.35)	0.024	1.22 (1.11, 1.34)	<0.001	1.31 (1.14, 1.49)	<0.001	1.28 (1.11, 1.48)	0.001
HH food security status								
Food secure	Reference		Reference		Reference		Reference	
Mildly food insecure	0.81 (0.64, 1.03)	0.081	1.02 (0.91, 1.15)	0.750	1.00 (0.84, 1.20)	0.980	1.01 (0.82, 1.25)	0.908
Moderately food insecure	0.96 (0.80, 1.14)	0.633	1.13 (1.03, 1.24)	0.012	1.13 (0.96, 1.33)	0.152	1.20 (1.01, 1.43)	0.038
Severely food insecure	1.02 (0.82, 1.27)	0.856	1.18 (1.02, 1.35)	0.024	1.13 (0.94, 1.36)	0.185	1.23 (0.98, 1.54)	0.073
HH type								
Poor	Reference		Reference		Reference		Reference	
Very poor	1.08 (0.96, 1.22)	0.193	1.11 (1.01, 1.22)	0.034	1.11 (0.98, 1.25)	0.092	1.1 (0.97, 1.26)	0.148
HH size								
≤4	Reference		Reference		Reference		Reference	
>4	1.10 (0.94, 1.27)	0.225	1.17 (1.05, 1.31)	0.004	1.20 (1.04, 1.40)	0.016	1.17 (0.98, 1.40)	0.075
Type of latrine								
Improved	Reference		Reference		Reference		Reference	
Nonimproved	1.34 (1.17, 1.53)	<0.001	1.15 (1.05, 1.27)	0.004	1.19 (1.02, 1.39)	0.025	1.31 (1.12, 1.54)	0.001
Type of floor								
Improved	Reference		Reference		Reference		Reference	
Nonimproved	0.97 (0.83, 1.15)	0.761	1.14 (1.01, 1.28)	0.034	1.10 (0.91, 1.32)	0.328	1.00 (0.81, 1.24)	0.978
HH head education								
At least 1 year of formal education	Reference		Reference		Reference		Reference	
No schooling	1.20 (1.06, 1.35)	0.004	1.11 (1.02, 1.22)	0.017	1.13 (1.00, 1.27)	0.043	1.04 (0.90, 1.20)	0.604

*Note*: Union was adjusted for as a cluster.

Abbreviations: HH, household; MDD‐W, minimum dietary diversity for women; MUACZ, mid‐upper arm circumference‐for‐age *z*‐score.

In terms of maternal characteristics, an age ≥ 25 years [aOR: 1.12 (95% CI: 1.02, 1.23); *p* = 0.016], MUAC < 24 cm [aOR: 1.73 (95% CI: 1.59, 1.88); *p* < 0.001], not receiving visits ANC by a skilled service provider [aOR: 1.15 (95% CI: 1.03, 1.28); *p* = 0.013], not taking more rest during pregnancy [aOR: 1.12 (95% CI: 1.02, 1.23); *p* = 0.021], delivery not in a health care facility [aOR: 1.23 (95% CI: 1.12, 1.35); *p* < 0.001] and a MDD‐W of less than five food groups [aOR: 1.11 (95% CI: 1.02, 1.22); *p* = 0.02] were associated with a MUACZ < −1.27. Moreover, some household characteristics, including the very poor type [aOR: 1.11 (95% CI: 1.01, 1.22); *p* = 0.034], a household size > 4 [aOR: 1.17 (95% CI: 1.05, 1.31); *p* = 0.004], a nonimproved latrine [aOR: 1.15 (95% CI: 1.05, 1.27); *p* = 0.004], water and soap/ash unavailable within 30 feet of the toilet structure [aOR: 1.22 (95% CI: 1.11, 1.34); *p* < 0.001], a nonimproved floor [aOR: 1.14 (95% CI: 1.01, 1.28); *p* = 0.034] and the head of the household with no schooling [aOR: 1.11 (95% CI: 1.02, 1.22); *p* = 0.017] were also associated with a MUACZ < −1.27. Children from mildly [aOR: 1.02 (95% CI: 0.91, 1.15); *p* = 0.75], moderately [aOR: 1.13 (95% CI: 1.03, 1.24); *p* = 0.012] and severely food insecure households [aOR: 1.18 (95% CI: 1.02, 1.35); *p* = 0.024] were also more likely to be malnourished based on a MUACZ < −1.27 compared with children from food secure households. Finally, we calculated the associated factors with wasting (WLZ < −2), MUAC < 12.5 cm and MUACZ < −2 using same multiple logistic regression analysis. From this model, we found that direction of strength of association for WLZ < −2 and MUACZ < −1.27 were similar but the status for MUAC < 12.5 cm and MUACZ < −2 was opposite for some important predictors, such as age and sex (Table 2).

## 4 DISCUSSION

We assessed data generated from a large‐scale survey data to determine whether the MUACZ represents an effective nutritional indicator and to identify the optimal cut‐off point to distinguish between nonwasting and wasting children. Our results demonstrate a strong correlation between WLZ and MUACZ, similar to an earlier study (Stephens et al., 2018). When wasting was defined using the WLZ as the gold standard indicator, the optimal cut‐off value for the MUACZ was −1.27; this cut‐off had a high sensitivity (i.e., classifies nonwasted children as healthy) and specificity (i.e., classifies wasted children as malnourished). The area under the ROC curve was almost 88%, indicating that this MUACZ cut‐off value exhibits excellent performance as an indicator of childhood malnutrition (Mandrekar, 2010). On the other hand, the sensitivities of the indicators MUAC < 12.5 cm and MUACZ < −2 were high but the values of specificities were less than 50%, which was not expected.

Some of the factors identified to be associated with MUACZ through multiple logistic regression are the most critical factors associated with child malnutrition (Choudhury et al., 2017; Rahman & Chowdhury, 2007; Vilcins et al., 2018). Our findings imply that age and sex are two key unmodifiable related factors, with older and male children being more wasted, which is consistent with earlier studies (Choudhury et al., 2017; Harding et al., 2018; National Institute of Population Research and Training (NIPORT) & ICF, 2020). MUAC < 12.5 cm, on the other hand, produced data with the opposite dimension, revealing the primary flaw in these indicators. Thus, the age‐ and sex‐specific indicator is most appropriate. But if we use MUACZ < −2, then the specificity was very low, whereas MUACZ < −1.27 gave expected and equal sensitivity and specificity.

Based on our ROC curve analysis and the associations with several factors, we suggest that a MUACZ < −1.27 represents a useful, simple nutritional indicator of malnourished children. A previous study showed that MUACZ is a good indicator of children's nutritional status as there is a linear relationship between MAUCZ and children's nutritional status (WLZ and body mass index) (Stephens et al., 2018). Another study evaluated the effectiveness of MUAC tapes by nonmedical volunteers in a community setting and reported that MUACZ effectively recognized severe acute malnutrition and moderate acute malnutrition (Miller et al., 2019).

However, MUAC can be easily measured in these cases, as only a measuring tape is needed to measure MUAC and then only a *z*‐score calculation is required to identify the nutritional status of the children (Rasmussen et al., 2012). Indeed, measurement of MUAC would be especially helpful when large numbers of children need to be screened quickly, particularly in humanitarian aid settings.

However, if MUAC < 12.5 cm is used as a cut‐off value to regress the outcome, then our regression analysis showed different results for age‐ and sex‐specific outcomes, which is not similar to previous study findings where the outcome variable is wasting (Choudhury et al., 2017). Whereas if MUACZ is being used for identifying acute malnutrition, then it gives a similar result as WLZ/WHZ does. Therefore, MUACZ might be a more appropriate indicator to identify wasting status. A previous finding revealed that MUAC is a good predictor of malnutrition (under‐ and overnutrition) in Sri Lankan schoolchildren, with different MUAC cut‐off values for malnutrition depending on age group and birthweight. The literature also confirms that when it came to predicting undernutrition, MUAC was more accurate in identifying thinness compared to stunting (Shinsugi et al., 2020).

Generally, a child's length and weight data limit the scope of estimating a child's nutritional status, especially if it happens to be a remote area. Moreover, length and weight data require height and weighing scales with high precision, and transporting these instruments is an added challenge. Alternatively, MUAC can be easily measured in these cases, as only a measuring tape is needed to measure MUAC and then only a *z*‐score calculation is required to identify the nutritional status of the children. According to prior findings, the best diagnostic indicators for undernutrition in children with diarrhoea are MUACZ. As children with diarrhoea are only assessed once before being sent home with ORS sachets, it may not be possible to reevaluate their nutritional status following rehydration in community‐based settings. In these circumstances, MUACZ can be used to effectively measure nutritional status, allowing for prompt diagnosis and commencement of community‐based nutritional supplements without the need for the child to return after rehydration for a repeat nutritional evaluation (Modi et al., 2015).

To reduce existing health disparities and boost the quality of care, mobile health (mhealth), as a potential tool for health care, has gained considerable attention in recent years. Globally, the use of mobile phones as a platform for health care delivery is gaining significant popularity and Bangladesh is no exception (Ahmed et al., 2014). Around 94% of households in Bangladesh have access to a mobile phone (NIPORT & ICF, 2020). Moreover, mhealth is also becoming a popular strategy to reach remote areas of Bangladesh (Khatun et al., 2014). In this context, our study findings may be used to develop a mobile app, whereby health care providers or any household member can measure the MUAC of children and input the measured data with date of birth and sex in the app. In this way, the nutritional status of the children can be easily identified by using this app.

### 4.1 Strength and limitations

We only covered extremely poor households in this study but we are confident of our study findings because of using an appropriate sampling method and a large sample size. Moreover, highly precise instruments were used to measure weight and length data. The field research personnel employed as data collectors underwent long‐term training. Nevertheless, there are some limitations to this study. The limitation was that we could not calculate MUACZ in under 3‐month‐old children since the WHO Anthro application gives us the MUACZ values for only 3‐month‐ to 5‐year‐old children, whereas the prevalence of wasting under 3‐month‐old children was 4%.

## 5 CONCLUSION

We conclude that a child's MUACZ is closely correlated with WLZ and appears to accurately identify childhood malnutrition, as defined by a WLZ < −2. Due to its simplicity and ease of use, a MUACZ < −1.27 may be considered an effective alternative to WLZ < −2 for detecting acute malnutrition among children aged 3–23 months. Thus, these findings enhance the evidence base to support the use of MUACZ as another nutritional indicator for childhood malnutrition.

## AUTHOR CONTRIBUTIONS

Tahmeed Ahmed and Nuzhat Choudhury originated the idea for the study and led the protocol design. Md Ahshanul Haque, Nuzhat Choudhury and Abu Syed Golam Faruque conceptualized the manuscript. S. M. Tanvir Ahmed, Sheikh Shahed Rahman, Mohammad Jyoti Raihan, Md Ahshanul Haque, Nuzhat Choudhury, Fahmida Dil Farzana and Tahmeed Ahmed contributed on survey design. Md Ahshanul Haque performed the statistical analysis and drafted the manuscript. Nuzhat Choudhury and Abu Syed Golam Faruque supervised the work, and critically reviewed and provided feedback for revising the manuscript. Nuzhat Choudhury oversaw the statistical analysis and suggested necessary improvements from a statistical point of view as well as public health perspective. Md Ahshanul Haque, Nuzhat Choudhury, Mohammad Jyoti Raihan, Mohammad Ali, Fahmida Dil Farzana, Farina Naz, Towfida Jahan Siddiqua, Abu Syed Golam Faruque, Tahmeed Ahmed and Sheikh Shahed Rahman contributed to the revision of the final draft for submission. All authors are responsible for the final content of the manuscript.

## CONFLICT OF INTEREST

The authors declare no conflict of interest.

## Data Availability

The data that support the findings of this study are available on request from the corresponding author. The data are not publicly available due to privacy or ethical restrictions.
